# XPO1 inhibition by selinexor induces potent cytotoxicity against high grade bladder malignancies

**DOI:** 10.18632/oncotarget.26179

**Published:** 2018-10-02

**Authors:** Han Bit Baek, Alan P. Lombard, Stephen J. Libertini, Aleida Fernandez-Rubio, Ruth Vinall, Regina Gandour-Edwards, Rachel Nakagawa, Kathleen Vidallo, Kristine Nishida, Salma Siddiqui, Hiromi Wettersten, Yosef Landesman, Robert H. Weiss, Paramita M. Ghosh, Maria Mudryj

**Affiliations:** ^1^ Veterans Affairs-Northern California Health Care System, Mather, CA, USA; ^2^ Department of Medical Microbiology and Immunology, University of California Davis, Davis, CA, USA; ^3^ Biochemistry, Molecular, Cellular, and Developmental Biology Graduate Group and Biotechnology Program, University of California Davis, Davis, CA, USA; ^4^ California Northstate College of Pharmacy, Elk Grove, CA, USA; ^5^ Department of Pathology and Laboratory Medicine, University of California Davis, Sacramento, CA, USA; ^6^ Department of Internal Medicine, University of California Davis, Davis, CA, USA; ^7^ Karyopharm Therapeutics, Newton, MA, USA; ^8^ Department of Urology, University of California Davis, Sacramento, CA, USA

**Keywords:** bladder cancer, XPO1, retinoblastoma, selinexor, cell cycle

## Abstract

Treatment options for high grade urothelial cancers are limited and have remained largely unchanged for several decades. Selinexor (KPT-330), a first in class small molecule that inhibits the nuclear export protein XPO1, has shown efficacy as a single agent treatment for numerous different malignancies, but its efficacy in limiting bladder malignancies has not been tested. In this study we assessed selinexor-dependent cytotoxicity in several bladder tumor cells and report that selinexor effectively reduced XPO1 expression and limited cell viability in a dose dependent manner. The decrease in cell viability was due to an induction of apoptosis and cell cycle arrest. These results were recapitulated in *in vivo* studies where selinexor decreased tumor growth. Tumors treated with selinexor expressed lower levels of XPO1, cyclin A, cyclin B, and CDK2 and increased levels of RB and CDK inhibitor p27, a result that is consistent with growth arrest. Cells expressing wildtype RB, a potent tumor suppressor that promotes growth arrest and apoptosis, were most susceptible to selinexor. Cell fractionation and immunofluorescence studies showed that selinexor treatment increased nuclear RB levels and mechanistic studies revealed that RB ablation curtailed the response to the drug. Conversely, limiting CDK4/6 dependent RB phosphorylation by palbociclib was additive with selinexor in reducing bladder tumor cell viability, confirming that RB activity has a role in the response to XPO1 inhibition. These results provide a rationale for XPO1 inhibition as a novel strategy for the treatment of bladder malignancies.

## INTRODUCTION

Bladder cancer is expected to account for ∼17,240 deaths in the US in 2018 [1]. Bladder cancers can be stratified as non-muscle invasive bladder cancer (NMIBC), which is confined to the mucosa, and muscle invasive bladder cancer (MIBC). While the majority of NMIBCs respond well to therapy (typically transurethral resection followed by BCG intravesical therapy), MIBC have proved more difficult to treat [2]. Cisplatin remains a key component of both GC (gemcitabine and cisplatin) or the MVAC (methotrexate, vinblastine, doxorubicin, and cisplatin) chemotherapy regimens but disease recurrence leading to death is common [3]. The recent identification of PD-1/PD-L1 inhibitors as an effective therapy for MIBC patients is the first addition to the armamentarium for the treatment of this malignancy in many years [4]. Identifying additional alternative treatments is critical for reducing the morbidity and mortality in patients with this disease.

Exportin-1/chromosome region maintenance 1 (XPO1/CRM1, referred to hereafter as XPO1), a well-characterized nuclear export protein, is involved in the export of many proteins and RNAs from the nucleus [5, 6]. In non-transformed cells the import and export of proteins is regulated through various processes and dysregulation of nuclear transport has been associated with carcinogenesis. XPO1 is instrumental in the nuclear export of numerous tumor suppressors and cell cycle regulators including retinoblastoma (RB). It is thought that excessive nuclear export may be a factor in the development of cancer and resistance to chemotherapy [7].

The RB family of pocket proteins, RB, p107 and p130, interact with E2F transcription factors to repress transcription [8, 9]. Phosphorylation of pocket proteins during cell cycle progression results in their dissociation from the E2Fs allowing them to function as transcriptional transactivators [8]. Deregulation of RB by mutations, deletions, epigenetic silencing, hyperphosphorylation or mis-localization is a common feature of malignancies [10]. Hyperphosphorylation of RB can be due to increased cyclin levels, particularly cyclin D, which in complex with cyclin dependent kinase (CDK) 4/6 predominantly targets RB. Similar alterations in p107 and p130 are less common [11], but several studies, including ours, reported a mislocalization of p130 to the cytoplasm in some tumors [12–14].

Bladder malignancies harboring RB mutations, including loss of heterozygosity, were reported almost 30 years ago [15, 16]. Reintroduction of RB into bladder cancer cells reduced tumor formation frequency [17] decreased cell proliferation, and was prognostic for poor outcome [18]. Subsequent studies reported that RB hyperphosphorylation resulted in inactivation of this tumor suppressor pathway [19]. A more recent study of 413 high grade muscle invasive bladder malignancies found that the RB gene, RNA or protein expression was altered in 17% of the malignancies [20]. Bladder malignancies with alterations in the RB and p53 pathways have been noted to have high proliferation rates [21]. In addition to a mutation, deletion or epigenetic silencing of the RB gene, the RB tumor suppressive pathway can be circumvented by overexpression of cyclin D, Cyclin D which along with its binding partners CDK4/6 hyperphosphorylates RB, thus disabling its tumor suppressive activity. Alternatively, CDK inhibitor deletions or mutations have similar effects, where cyclin/CDK activity, and subsequently RB phosphorylation is elevated. While mutated or deleted RB cannot be replaced, preclinical studies utilizing CDK4/6 inhibitors have shown that these compounds have efficacy in retarding growth of certain bladder tumors in an RB dependent manner [22].

Previous reports show that selinexor (KPT-330), a selective inhibitor of nuclear export (SINE) is an effective and specific inhibitor of XPO1 and a putative new therapeutic for the treatment of multiple malignancies [23]. *In silico, in vitro* and *in vivo* studies reported here show that XPO1 is expressed in most bladder malignancies, and that selinexor effectively reduces XPO1 expression and cell viability in a dose dependent manner in all cells. Mechanistic studies reveal that the drug induces cell cycle arrest and apoptosis, and that the RB/E2F network is a component of the response to selinexor. These studies show that this drug may be an effective strategy for inhibiting MIBC tumor growth.

## RESULTS

### XPO1 is elevated in bladder tumor cells

**Figure 1:**
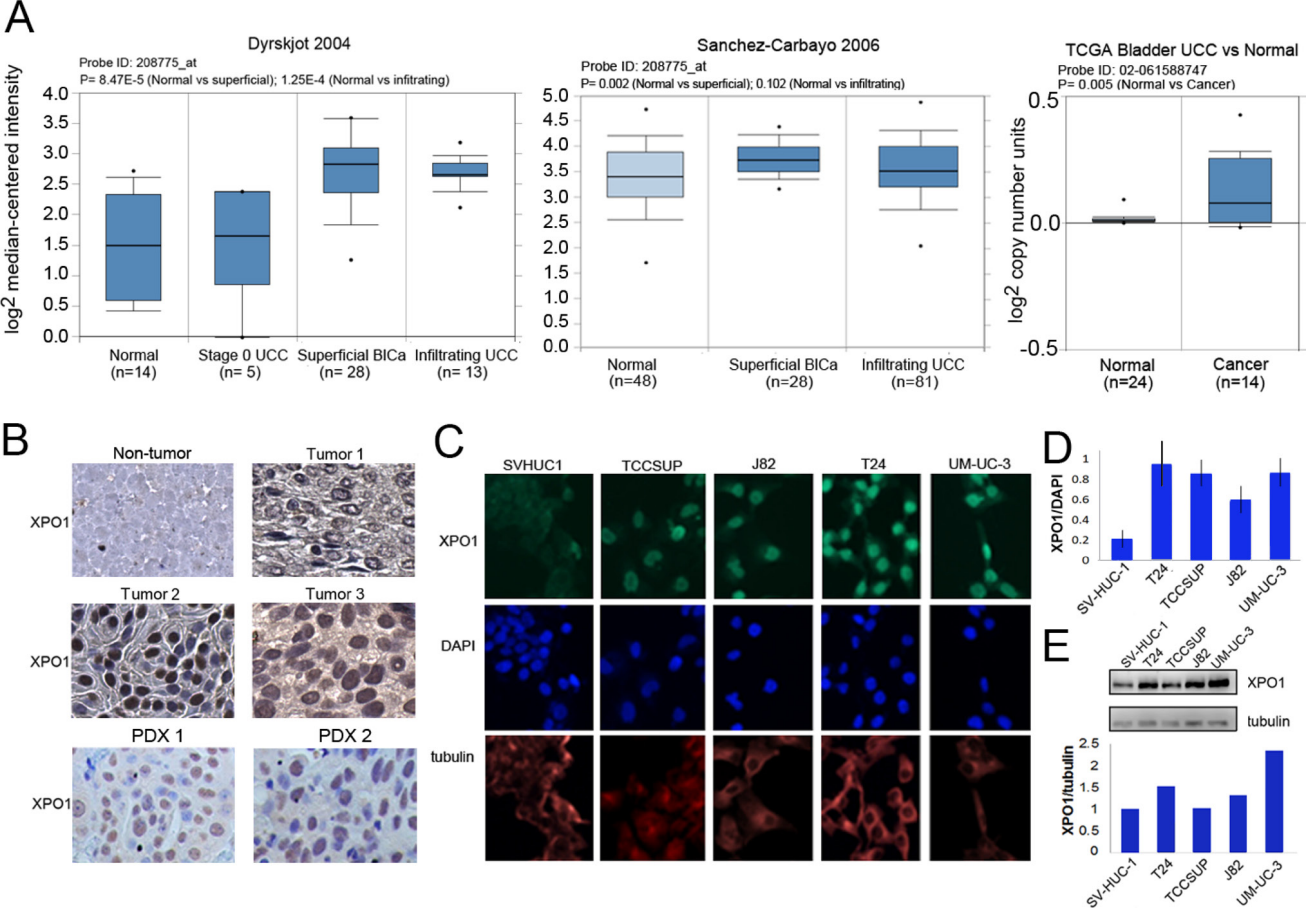
Expression of XPO1 in bladder tumor cells (**A**) XPO1 expression in normal and bladder cancer from the indicated ONCOMINE datasets. The top and bottom of the box indicates the 75th and 25th percentile, respectively. Number of samples (*n*) and *p*-values (determined by a two-tailed Mann-Whitney *U* test) are as shown. (**B**) Representative images of XPO1 IHC staining of primary high-grade bladder malignancies (upper panels) and two PDX tumors. 40× magnification. Cells were counter-stained lightly with H&E. (**C**) Immunofluorescent analysis of XPO1 expression (green) in bladder tumor cells, where tubulin staining (red) and DAPI staining (blue) served to define the cytoplasmic and nuclear compartments, respectively. The analyses were conducted at the same time with the same reagents. (**D**) Quantification of immunofluorescence where XPO1 levels were normalized to DAPI. (**E**) Western immunoblot analysis of XPO1 expression, where tubulin served as a loading control (upper panel). Normalization of XPO1 expression to tubulin (lower panel, intensity to XPO1/intensity of tubulin). The studies were repeated at least once. Error bars denote standard deviation. Student’s *t* test.

A review of Oncomine datasets identified two studies which showed highly statistically significant increases of XPO1 expression in bladder tumors when compared with control tissue (Figure 1A). Additionally, TCGA (The Cancer Genome Atlas) data, indicated that there was an increase in XPO1 gene copy number in cancer tissue. There are three additional studies reported on Oncomine where XPO1 transcripts are elevated, and *P* values trended toward significance (0.058 to 0.102). There is one study that shows no significant increase in XPO1 levels. Taken together the data indicate that there is an increase in XPO1 expression in bladder malignancies.

To assess XPO1 protein levels in clinical tumor samples, archival MIBC tumor tissues were used to construct a tissue array. The bladder tumor tissue array consisting of 53 high grade urothelial carcinomas was used to determine XPO1 expression (Table 1). The age of the bladder cancer patients ranged from 36 to 85 with an average of 65.7. There was a marked gender disparity where 44 of the tumors were from males and 9 were from females. Most of the tumors were urothelial carcinomas and all tumors were high grade. XPO1 staining was detected in the nucleus and in the cytoplasm. There was a variation in the intensity of staining in these compartments between tumor samples. Previous studies reported that XPO1 can be present in the nucleus and the cytoplasm [24, 25]. In Figure 1B, tumor 1 is representative of tumors with low levels of cytoplasmic and nuclear staining. Tumor 2 represents tumors with intense nuclear staining, but minimal cytoplasmic staining. Tumor 3 represents tumors with strong XPO1 staining in most nuclei and cytoplasm. Staining was not detected in non-tumor bladder tissue. In robustly staining cells, XPO1 was predominantly nuclear. High levels of XPO1 staining were detected in one or both compartments in 70% of the tumors, while low levels were detected predominantly in the cytoplasm in 30% of the tumors. There was no correlation between XPO1 expression and gender, age, tumor type or stage. We accessed XPO1 expression in 13 bladder tumor patient derived xenografts generated from high grade malignancies that were established by our colleagues at UC Davis [26]. Nuclear XPO1 staining was detected in all samples (Figure 1B).

**Table 1:** Patient and tumor characteristic

	Number	XPO1 medium/high	XPO1 low
**Total**	**53**	**38**	**15**
**Gender**			
Female	9	6	3
Male	44	32	12
**Age range: 36–85; average: 65.7**			
under 60	16	11	5
60–69	13	10	3
70 and over	25	17	8
**Race**			
not listed	17	12	5
African American	1	1	0
Asian	1	0	1
Caucasian	34	25	9
Caucasian Hispanic	4	4	0
Caucasian non Hispanic	30	21	9
**Tumor type**			
not reported	1	1	0
Papillary urothelial carcinoma	13	9	4
SCC	1	1	0
Urothelial Carcinoma	38	27	11
**Grade**			
High grade	50	35	15
Low grade	2	2	0
Unknown	1	1	0

XPO1 protein levels were assessed in 4 MIBC tumor derived cell lines (T24, TCCSUP, J82, and UM-UC-3) and in a non-transformed but immortalized bladder epithelial cell line (SV-HUC1) by immunofluorescence (IF) and western blot analyses (Figure 1C and 1D). Immunofluorescence studies showed that XPO1 expression was predominantly nuclear but were also detected in the cytoplasm. The intensity of XPO1 staining was normalized to DAPI (Figure 1D). Western blot analyses (Figure 1E) were also used to assess XPO1 protein levels, where expression was normalized to tubulin. Both indicate that XPO1 expression is lowest in SV-HUC-1 cells. Not unexpectedly, normalization to DAPI and tubulin give somewhat different results. This is most likely due to differing levels of tubulin in the different cells.

### Selinexor reduces XPO1 expression and attenuates bladder tumor cell viability

**Figure 2:**
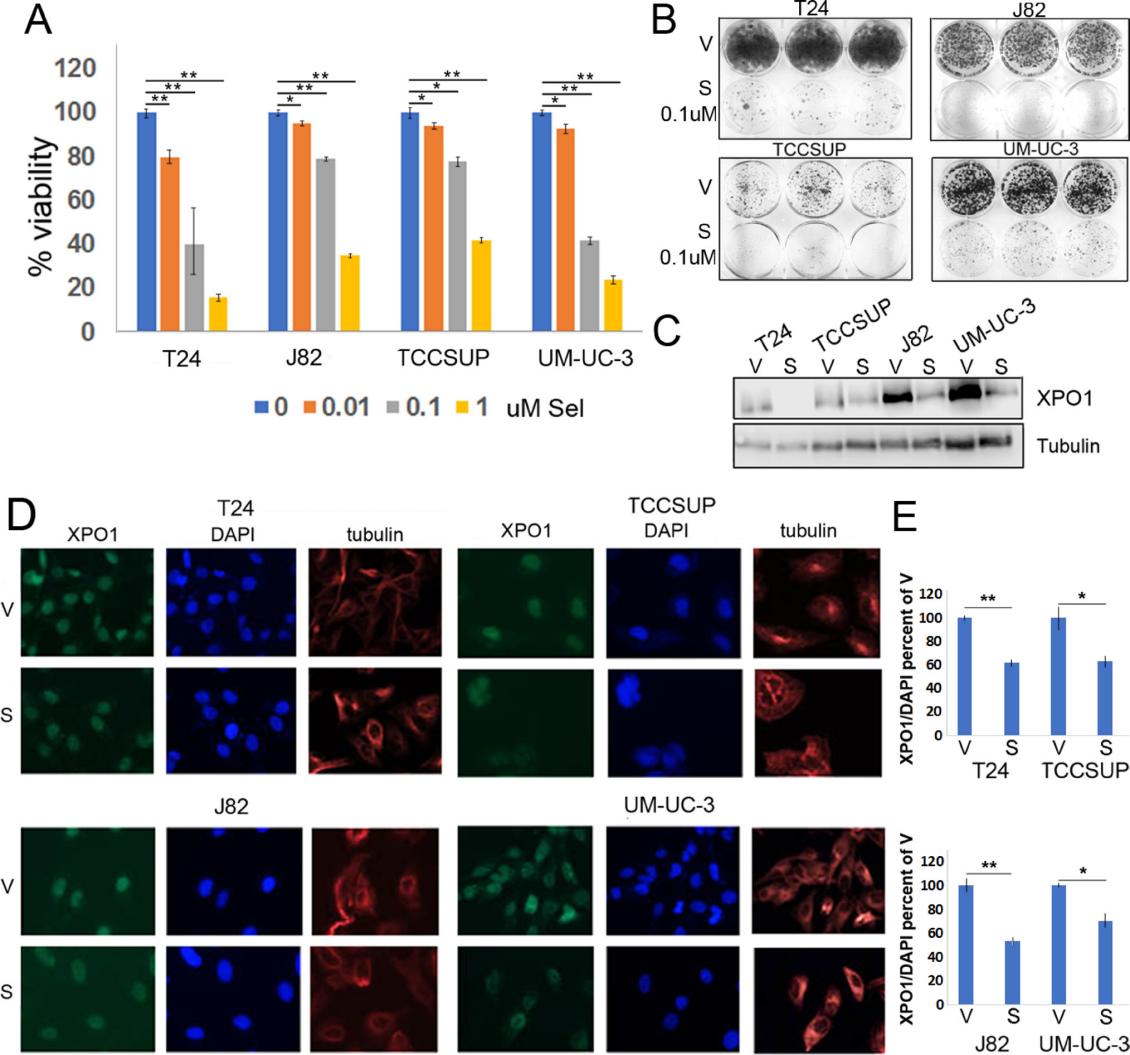
Selinexor reduces bladder tumor cell the viability (**A**) Cell viability assays of four bladder tumor cell lines treated with different concentrations of selinexor for 72 hours. Error bars denote standard deviations. (**B**) Colony formation assays of four cell lines treated with 0.1 uM selinexor or vehicle for T24-12 days, UM-UC-3-11 days, J82-9 days and TCCSUP-10 days. At the end of the study, the cells were fixed with methanol and stained with crystal violet. (**C**) Expression of XPO1 in bladder tumor cell lines treated with 0.1 uM selinexor for 72 hours analyzed by Western immunoblots. Tubulin serves as a protein loading control. V = vehicle, S = selinexor. (**D**) Immunofluorescent detection of XPO1 (green) in cells treated with vehicle and selinexor. Cells were plated on chamber slides and they were treated with either vehicle or selinexor for 48 hours. Tubulin (red) and DAPI (blue) served to define the nuclear and cytoplasmic compartment, respectively. (**E**) Quantitation of XPO1 expression normalized to DAPI. ^*^denotes *p* ≤ 0.05, ^**^denotes *p* ≤ 0.01. S.E.M.

Previous studies have reported that selinexor interacts with XPO1, which results in a reduction of XPO1 levels and in tumor cell viability [27]. Dose response studies were conducted to define the sensitivity of various bladder tumor cells to selinexor. Cells were treated with vehicle or varying concentrations of selinexor for 72 hours. In all lines tested, cell viability decreased in a dose dependent manner (Figure 2A). Selinexor was particularly effective in reducing cell viability of T24 and UM-UC-3 cells where 0.1 uM reduced cell viability by ∼50%. The longer-term colony formation assays complimented the CCK-8 assay and confirmed cell sensitivity to selinexor (Figure 2B). To assess the effect of selinexor on XPO1 levels, cells were treated with vehicle (DMSO) or 0.1 uM selinexor for 72 hours and subjected to IF or western immunoblots analyses. IF studies showed a decrease in XPO1 levels in all cell lines (Figure 2D and 2E) and western immunoblot analysis confirmed these results (Figure 2C).

### Selinexor induces a cell cycle arrest and apoptosis

**Figure 3:**
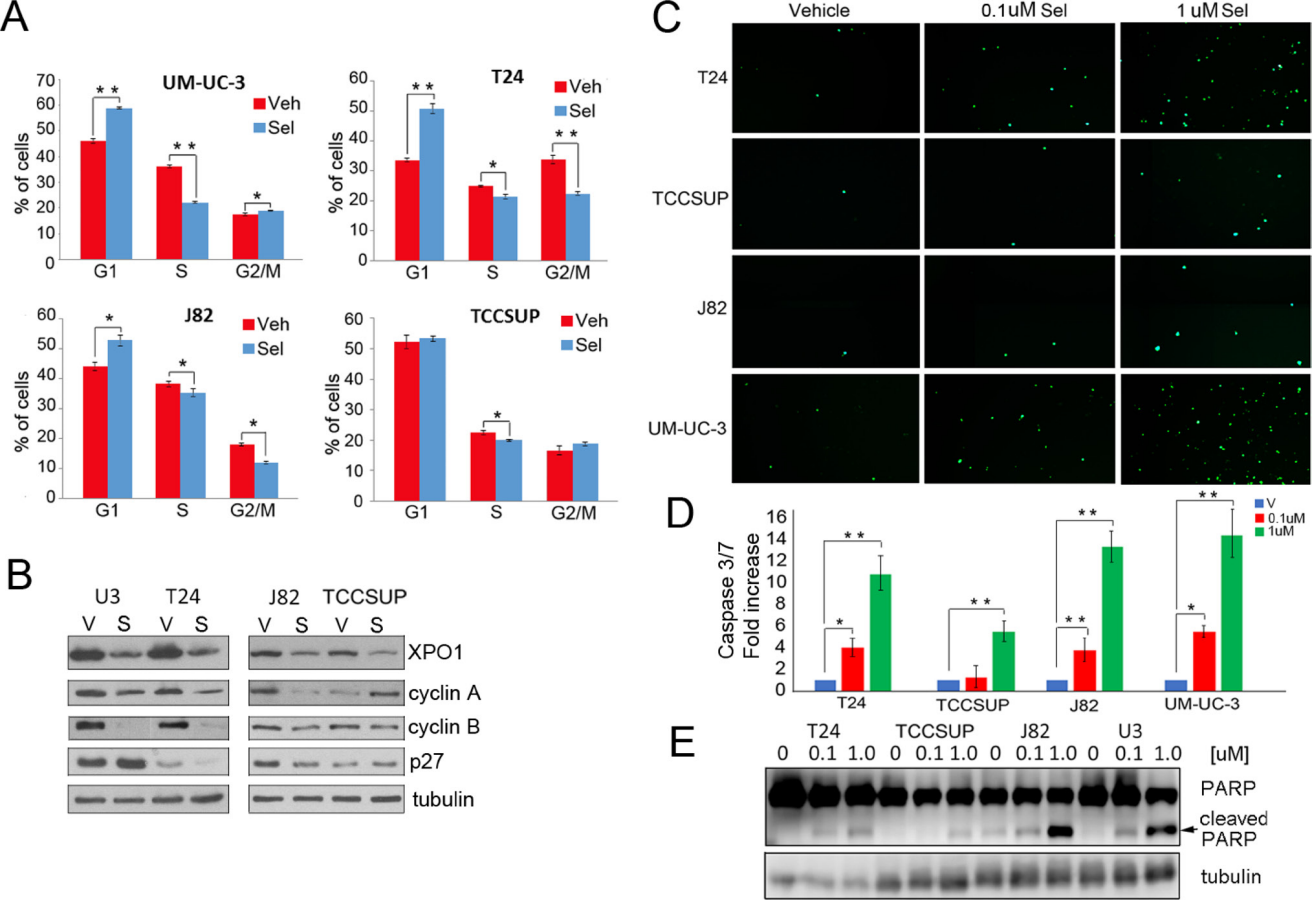
Selinexor induces cell cycle arrest and apoptosis (**A**) Cell cycle analysis of selinexor treated cells was assessed by flow cytometry. Cells were treated with vehicle (red) or 0.1 uM selinexor (blue) for 72 hours. (**B**) Expression of XPO1, cyclin A, cyclin B, and p27 in cells treated with vehicle (V) or 0.1uM selinexor (S) was assessed by Western immunoblot studies. Tubulin served as a loading control. (**C**) Cells were treated with two different doses of selinexor for 72 hours and followed by caspase3/7 immunofluorescence assays. (**D**) The extent of apoptosis detected by the caspase 3/7 assay was quantified by counting 4 separate fields and shown as fold increase over control. (**E**) Detection of PARP cleavage in cells treated with vehicle or increasing doses of selinexor for 72 hours by Western blot analysis. Arrow points to cleaved PARP. Tubulin served as a loading control. ^*^denotes *p* ≤ 0.05, ^**^denotes *p* ≤ 0.01. Error bars denote standard deviation; Student’s *t* test.

While viability studies indicated that selinexor reduces cell viability, the analyses did not differentiate between a cell cycle arrest and an increase in apoptosis. Flow cytometry was used to determine the effect of selinexor on cell cycle (Figure 3A). Cells were treated with vehicle or 0.1 uM selinexor for 72 hours, fixed, stained with propidium iodide, and subjected to flow cytometry. All cell lines except TCCSUP exhibited a G1 arrest. A statistically significant decrease in S phase was apparent in all cells. Moreover, T24 and J82 cells also exhibited a decrease in G2/M phase. Hence, XPO1 inhibition retards cell cycle progression. Western immunoblot studies show that selinexor treatment elevates p27 expression in some, but not all cells. Furthermore, selinexor treatment decreases cyclin A and cyclin B expression in all but TCCSUP cells. Elevation of p27 and reduction of S phase cyclins is consistent with a decrease in proliferation. (Figure 3B).

Two different methods were used to determine if selinexor induces an apoptotic response. Cells treated with vehicle or selinexor for 72 hours were subjected to a cleaved caspase 3/7 immunofluorescent assay. All cell lines exhibited a dose-dependent increase in caspase 3 and 7 cleavage (Figure 3C and 3D). Western immunoblot analysis was conducted to detect PARP cleavage, a well-documented marker of apoptosis (Figure 3E). Dose dependent increases in cleaved PARP were evident in all cell lines treated with the drug. Notably, selinexor induced apoptosis at the 0.1 uM concentration in all cells except TCCSUP. Taken together, the data demonstrates that selinexor elicits a growth arrest and an apoptotic response in several models of bladder cancer.

**Figure 4:**
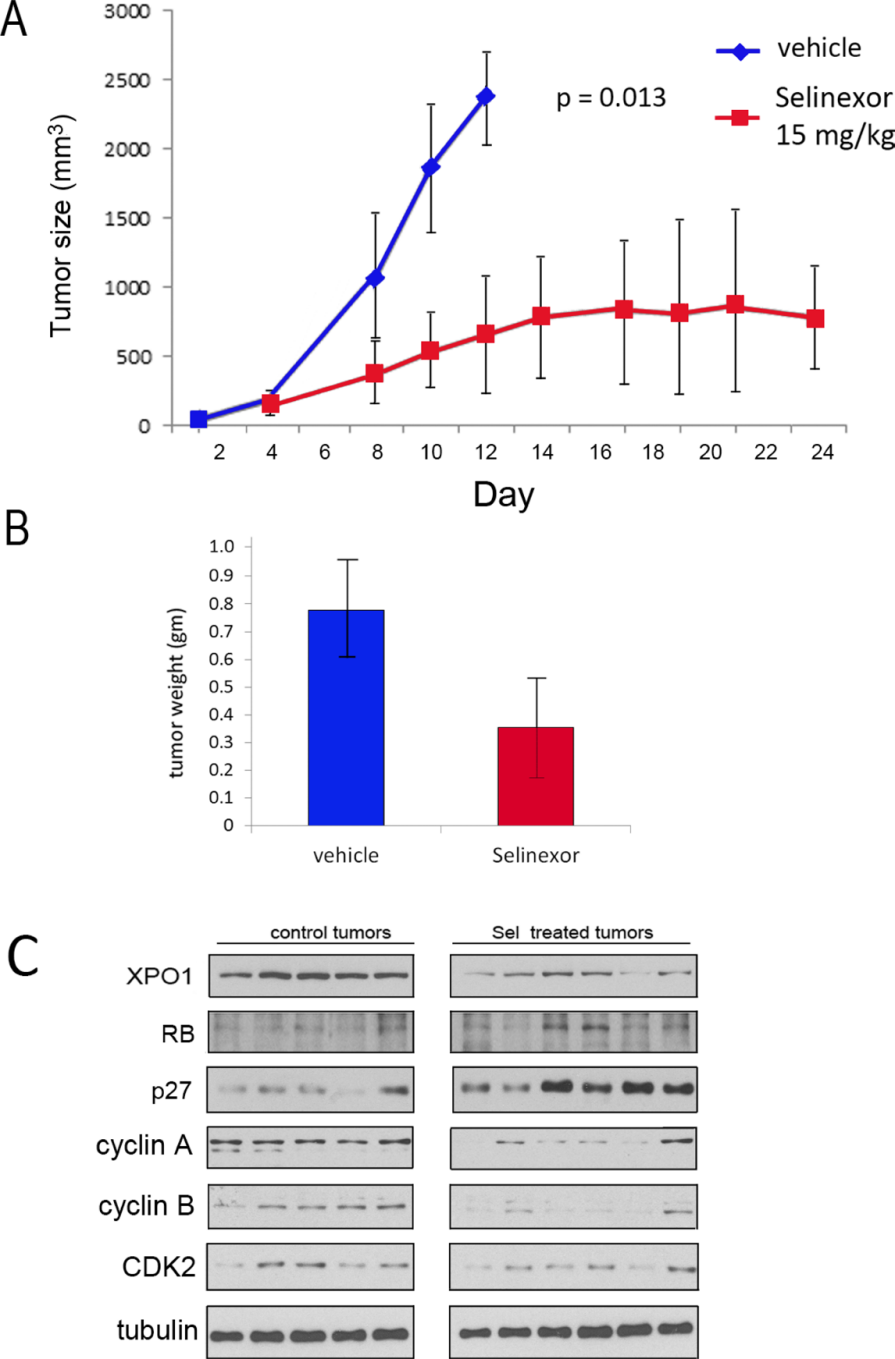
UM-UC-3 cell xenograft growth is retarded by Selinexor (**A**) 10^6^ UM-UC-3 cells were re-suspended 1:1 in Matrigel:PBS and injected into the left flank of female athymic nude mice. 5 mice were treated with vehicle and 7 were treated with selinexor 15 mg/kg 3× per week. Tumors were measured using calipers and volumes were calculated using the formula tumor volume (mm^3^) = length × width × depth. Mean tumor volume ± standard deviation. The difference in tumor growth rates were statistically significant. (**B**) Following euthanasia, tumors were excised and weighed (vehicle treated = 5 tumors, selinexor treated 6 tumors). The difference in weight was statistically significant *P* ˂ 0.05. Mean tumor weight ± standard deviation. Student’s *t* test. (**C**) Western immunoblot analysis of vehicle and selinexor treated tumors extracts. Tubulin served as loading control.

The *in vivo* efficacy of selinexor was assessed in a xenograft study using UM-UC-3 cells. Cells (10^6^) were implanted into the flank of athymic female mice. Once tumors were apparent, mice were treated with either vehicle (5 mice) or 15 mg/Kg of selinexor (7 mice) by oral gavage 3 times per week, a dosing schedule reported to be effective in reducing growth of other tumor types [28–30]. The growth rate for each tumor was followed 3 times per week over the course of the experiment. Treatment with selinexor significantly impaired tumor growth (Figure 4A; growth rate of each individual tumor and Kaplan–Meier survival data in Supplementary Figure 1). Mice were weighed twice per week and selinexor treated mice did not exhibit a weight loss, or any discernable adverse effects. After mice were euthanized the tumors were excised and flash frozen. The weight of tumors from selinexor treated mice was significantly smaller than the weight of tumors from vehicle treated mice (Figure 4B). One of the selinexor treated mouse tumors was too small to obtain sufficient tumor tissue for analysis. Western immunoblot analyses of tumor tissue extracts indicate that drug treatment reduced XPO1 expression (Figure 4C). Furthermore, elevation of RB and p27 and reduction of cyclin A, cyclin B, and CDK2 in selinexor treated tumors confirms that selinexor impairs tumor cell proliferation *in vivo*.

### XPO1 inhibition increases wildtype RB nuclear levels

Previous studies have shown that XPO1 is important for the nuclear export of several tumor suppressors including RB [31, 32]. Of the four bladder tumor cell lines studied, T24 and UM-UC-3 cells express wildtype (wt) RB [33]. RB is deleted in TCCSUP cells and is mutated and expressed at very low levels in J82 cells [33–37]. All four cell lines express RB related proteins, p107 and p130.

**Figure 5:**
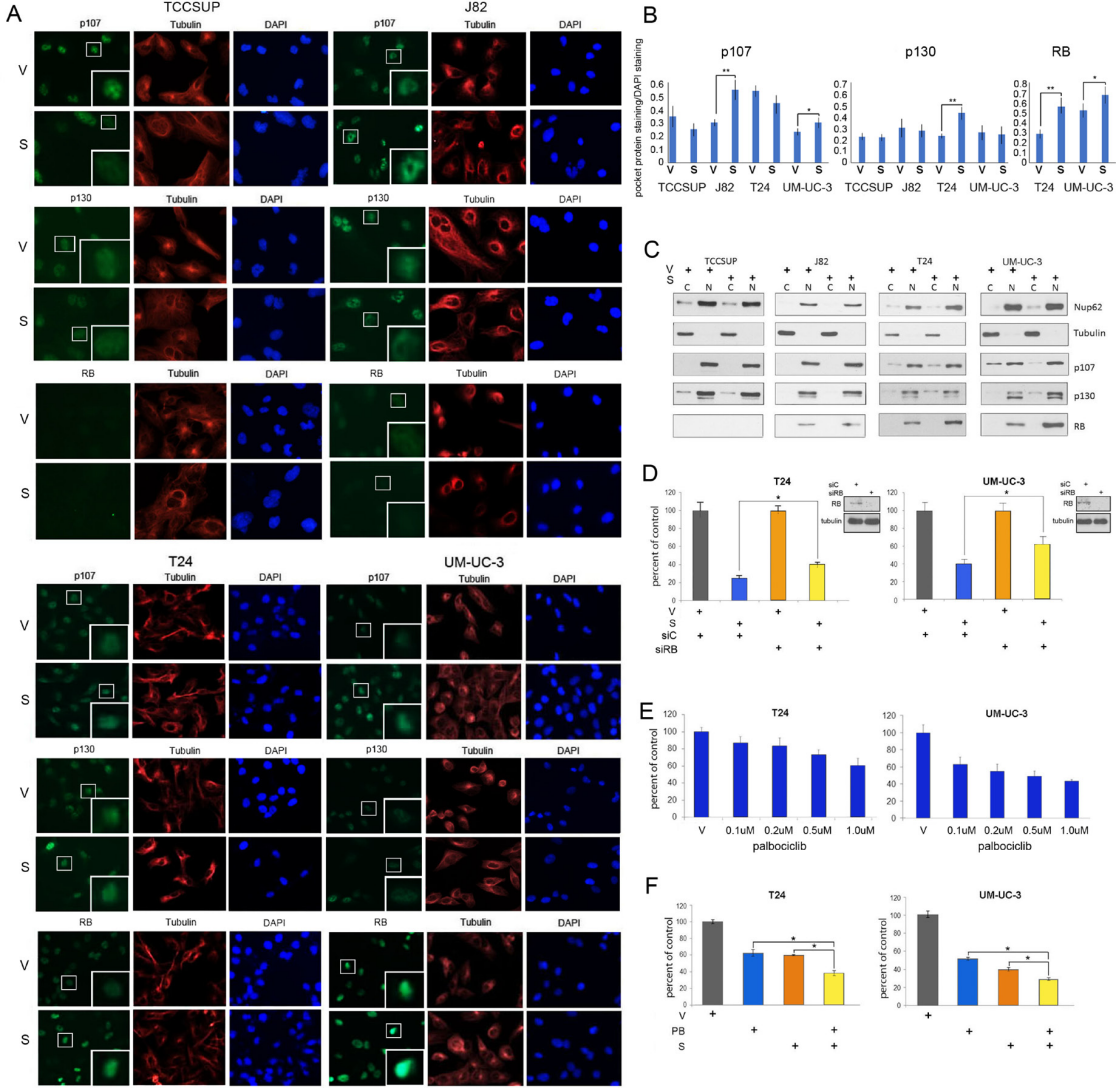
Selinexor alters pocket protein expression in nuclear and cytoplasmic compartments (**A**) Representative images of p107, p130 and RB (green) IF staining of bladder cancer cells treated with vehicle (V) or selinexor (S) for 48 hours. Tubulin staining (red) and DAPI staining (blue) served to define the cytoplasmic and nuclear compartments, respectively. The inserts are magnifications of the boxed cells. (**B**) Quantification of staining intensity of pocket proteins normalized to DAPI. (**C**) Nuclear and cytoplasmic fractions of cell treated with vehicle or 0.15 uM selinexor (UM-UC-3 and T24 cells), 0.25 uM selinexor (J82) and 0.5 uM selinexor (TCCSUP) for 72 hours were assessed for the expression of RB, p107 and p130. Nup62 and tubulin were used as markers for the nuclear and cytoplasmic fractions, respectively. (**D**) T24 and UM-UC-3 cells transfected with siC or siRB and were treated with vehicle or 0.1 uM selinexor for 72 hours. The results are shown as percent cell viability comparing drug treated to vehicle treated cells. (**E**) Palbociclib reduces T24 and UM-UC-3 bladder tumor cells viability in a dose dependent manner. (**F**) Combined selinexor (0.1 uM) and palbociclib (0.5 uM) treatment is more effective in reducing viability of cells than either treatment alone where the CI = 1.04 for UM-UC-3 cells and 1.02 for T24 cells indicating an additive response. Error bars = ± standard deviation. Student’s *t* test; ^*^ denotes *p* ≤ 0.05, ^**^ denotes *p* ≤ 0.01.

Two different techniques were used to study expression levels and localization of the pocket proteins. IF was used to analyze RB, p107 and p130 expression in cells treated with vehicle or selinexor. Co-staining with tubulin antibodies (red) and DAPI (blue) identified the cytoplasmic and nuclear compartments, respectively (Figure 5A). RB expression was exclusively nuclear whereas p107 and p130 expression was mostly nuclear and faintly cytoplasmic in some cells. Nuclear expression of p130 was affected by drug treatment in T24 cells. p107 expression was unchanged in TCCSUP and T24 cells but was elevated in J82 and UM-UC-3 cells. RB levels were extremely low in J82 cells. UM-UC-3 and T24 cells have higher levels of RB, which were further augmented by selinexor treatment. The expression levels of pocket proteins were quantified and standardized to DAPI staining (Figure 5B).

Subcellular localization of the pocket proteins was additionally evaluated by cell fractionation studies. Successful fractionation was confirmed by the analysis of tubulin, a cytoplasmic marker, and Nup62, a nuclear marker (Figure 5C). These studies show that RB expression is exclusively nuclear while p107 and p130 is mostly nuclear but detected in the cytoplasmic compartment in some cells. Treatment with selinexor increased nuclear levels of RB in T24 and UM-UC-3 cells. Selinexor did not affect mutant RB in J82 cells. There was a small increase in the nuclear levels of p107 and p130 in some cells, along with a shift in the ratios of cytoplasmic/nuclear levels of these proteins. The fractionation studies confirmed the IF results that RB levels are elevated by XPO1 inhibition.

### Modulation of RB modifies cellular response to selinexor

To test the hypothesis that wt RB plays a role in selinexor-mediated decrease in viability, T24 and UM-UC-3 cells were treated with either control or RB siRNA and subsequently treated with vehicle or 0.1uM selinexor for 72 hours (Figure 5D). Cell viability following drug treatment was scored as percent of vehicle treated cells. Depletion of RB curtailed the effect of selinexor treatment.

Wt RB tumor suppressive function can be impaired by cyclin D/CDK4/6-mediated phosphorylation. Therefore, inhibition of CDK4/6 results in decreased RB phosphorylation, enhanced RB binding to E2Fs and increased transcriptional repression. Since RB depletion reduced sensitivity to selinexor, augmenting hypophosphorylated levels of RB should enhance selinexor efficacy. The CDK4/6 inhibitor palbociclib has demonstrated efficacy in limiting growth of cells that express wt RB [38]. As expected, T24 and UM-UC-3 cells were sensitive to palbociclib in a dose-dependent manner (Figure 5E), while J82 and TCCSUP cells were not affected by this treatment (Supplementary Figure 2). T24 and UM-UC-3 cells were treated with vehicle alone, selinexor alone, palbociclib alone, or the combination of selinexor (0.1 uM) and palbociclib (0.5 uM) for 72 hours. Dual drug treatment of T24 and UM-UC-3 cells was significantly more effective (Figure 5F) than either single agent suggesting that this combination may be an effective treatment strategy for bladder malignancies that express wt RB.

## DISCUSSION

Malignancies, including bladder tumors, have deregulated expression and localization of multiple proteins that collectively promote proliferation and survival of transformed cells. Previous studies have demonstrated that targeting XPO1-mediated nuclear export, which is often augmented in malignancies, is an effective strategy to limit tumor cell growth [6, 23].

The current study focused on bladder malignancies and showed by data mining and tumor tissue array analysis that XPO1 is elevated in bladder tumor cells. Cultured bladder tumor-derived cells have higher XPO1 levels than immortalized SV-HUC1 cells. Bladder tumor cells are sensitive to selinexor in a dose dependent manner, where the IC_50_ dose ranged from 0.1–0.5 uM. Selinexor efficiently induced apoptosis and growth arrest. These results are consistent with previous studies, which showed that limiting XPO1 expression induces a growth arrest and an apoptotic response [27, 39–41]. The dose of selinexor that is sufficient to induce apoptosis and inhibit cell cycle progression is significantly lower than what was attained in phase 1B clinical trial [42], arguing that this drug may be a viable new treatment option for bladder malignancies. *In vivo* studies showed that selinexor was effective in reducing tumor growth as a single agent where tumors treated with selinexor exhibited a decreased growth rate, significantly smaller tumor volume at the termination of the study and altered expression of proteins that are consistent with growth arrest.

While all the bladder tumor cells tested were sensitive to selinexor treatment, T24 and UM-UC-3 cells, which express wt RB, exhibited a heightened response to XPO1 inhibition. The importance of XPO1 in RB transport is controversial. Initial studies concluded the RB was not an XPO1 cargo, but subsequently studies indicated that RB phosphorylation affected XPO1-mediated nuclear export [32, 43]. The role of XPO1 in trafficking of p107 and p130 has not been explored. Our immunofluorescence and cell fractionation studies addressed the effect of XPO1 inhibition on the expression level and localization of the pocket proteins. XPO1 inhibition has a modest affect in altering the abundance of p107 and p130 and a small effect in compartmental redistribution. In the bladder tumor cells studied RB expression was limited to the nucleus in both treated and untreated cells, but selinexor significantly increased the abundance of wt RB in the nucleus. It is unknown if limiting XPO1 results in RB accumulation due to decreased export from the nucleus or if other mechanisms are instrumental in elevating RB. A similar increase of nuclear p53 in thymic epithelial tumors was apparent following selinexor treatment [44]. The increase in wt RB was coincident with a decrease in cyclin A, a direct E2F/RB target, and cyclin B. These S phase cyclins are essential for cell cycle transversal and their decline is consistent with a proliferative arrest. Biochemical analyses of the resected tumors also showed that selinexor treated tumors had reduced XPO1 expression, increased RB and p27 levels and a decline of cyclin A, cyclin B and CDK2 levels, consistent with a growth arrest.

While RB-mediated growth arrest has been well studies, growth arrest can be RB-independent, as we observed for the RB mutant bladder cells TCCSUP and J82. There are several potential mechanisms of RB-independent growth arrest. An increase of CDK inhibitors p21 and p27 would impedes the activity of cyclin E, A and B, thus preventing cell cycle transversal regardless of RB status. Treatment of RB mutated cells with histone deacetylase inhibitor resulted in growth arrestand flavone treated RB–/– mouse embryo fibroblasts were able to undergo a growth arrest. Cells where RB, p107 and p130 were deleted were unable to respond in this manner, arguing the p107 and p130 also have a role in growth arrest [45].

The role of RB in apoptosis is far less explored, but recent interest in CDK4/6 inhibitors which rely on RB tumor suppressive function and induce an apoptotic response, has revealed that the mechanism is complex, but has been observed in different cellular contexts. In lung cancer cells, CDK4/6 inhibition results in suppression of IAPs, FOXM1 and survivin and an augmentation of SMAC and caspase 3 expression [46]. Induction of apoptosis following CDK4/6 inhibition in thyroid cancer cells involved a modulation of FOXM1, cyclin A1 and myc [47]. CDK4/6 inhibition in melanoma cells resulted in apoptosis associated with deregulation of BCL2, BCL2L1, BIRC5 and BIM [48]. These studies argue that while RB is instrumental in apoptosis, mechanisms are complex and cell dependent. Interestingly, we did not detect increased PARP cleavage in the drug treated tumors (data not shown). During the long-term treatment, the induction of apoptosis may result in cell death and elimination of the apoptotic cells while the remaining cells undergo a growth arrest.

The hypothesis that RB contributes to selinexor sensitivity was tested by modulating RB activity in two different ways in cells expressing wt RB. First, RB was ablated by siRNA. Decrease in RB levels reduced cellular sensitivity to selinexor. Second, RB activity was augmented by inhibiting CDK4/6-mediated RB phosphorylation using the CDK4/6 inhibitor palbociclib. Treatment of cells with palbociclib enhanced selinexor efficacy. Together these analyses argue that in this cellular context RB partly mediates the effect of selinexor. Our conclusions differ somewhat from previous studies. Studies conducted in sarcoma cells found that selinexor-mediated cell cycle arrest was independent of RB, p53 and p21 [49] since cells depleted of these proteins or with mutant forms of these proteins responded to selinexor treatment. In this study there was no increase in RB levels, but the authors reported a decrease in RB phosphorylation. In models of ovarian and breast cancers, researchers concluded that the response to XPO1 inhibition was independent of p53, RB, and FOXO [50]. In contrast, studies in other contexts reported that p53 and p21 are components of the selinexor response [51, 52]. Our results and the studies cited above can be reconciled since we have found that cells respond to selinexor in the absence of wt RB but have a more robust response in the presence of wt RB. Moreover, this effect was further enhanced by CDK4/6 inhibition. Since RB inactivating mutations are found in only 11-20% of bladder malignancies [2], most bladder tumor patients could benefit from a therapy that is at least partly reliant on the presence of wt RB. Moreover, deletions of CDK inhibitors CDKN2A and CDKN2B, each of which inhibit CDK4/6 activity occur in over 50% of bladder malignancies. Therefore most malignancies should be susceptible to CDK4/6 inhibition [53], and preclinical studies reported that CDK4/6 inhibition of RB positive bladder cancer cells was effective in limiting cell proliferation [38]. Our results suggest that the combined increase of nuclear RB and decrease of RB phosphorylation is an effective combination for limiting cell viability and is a potential therapeutic strategy.

## MATERIALS AND METHODS

### Cell culture and transfections

SV-HUC-1, T24, TCCSUP, J82, and UM-UC-3 cells were from American Type Culture Collection (ATCC) and used within 6 months of purchase. All cell lines were cultured in 10% FBS RPMI-1640 supplemented with glutamine and both penicillin and streptomycin at 37C and 5% CO_2_. ATCC used the following cell line authentication process: Short Tandem Repeat (STR) profiling. The 17 STR loci plus Amelogenin are amplified using Promega’s PowerPlex® 18D System. A comprehensive analysis report interprets both karyotypically normal and abnormal cell lines, including electropherograms supporting the allele calls at each locus, known reference profiling against the ATCC STR database and a comprehensive interpretation of results. The cells were tested for mycoplasma within the last 6 months using the Lonza Walkersville, Inc Mycoalert detection kit (LT07-218). All transfections were carried out using Lipofectamine 2000 (Invitrogen 11668-027) at a concentration of 50nM. The following oligonucleotides were used for transfection: siRB AACACCCAGGCGAGGUCAGAAUUUU (Dharmacon), and control non-targeting oligonucleotide (Dharmacon D-001810-10-50). The following drugs were used for treatments: palbociclib (in DMSO) (Selleckchem PD-0332991 and selinexor which was obtained from Karyopharm and dissolved in DMSO.

### Tissue microarray

After institutional review board approval, the pathologist (R.G.E.) reviewed the histologic sections and selected appropriate paraffin blocks. The samples were de-identified. A paraffin tissue microarray block was constructed of triplicate 60-um core samples using the Beecher Instruments Manual Tissue Arrayer. The pathologist (R.G.E.) identified areas of appropriate tissue on the corresponding histologic sections. Then, 60-um core samples were extracted from the specific areas of the donor blocks and inserted in the array block. A hematoxylin-eosin section was prepared and used as a reference to interpret the immunostained sections. The bladder tumor tissue array consisted of 53 high grade tumors. Each tumor was arrayed in triplicate. The bladder PDX tissue array was provided by the UCD Cancer Center pathologist (R.G.E.) and the generation of the PDX tumors was previously reported [26].

### Immunohistochemistry

Paraffin-embedded blocks were sectioned and rehydrated in xylene and graded alcohols. Antigen retrieval was performed with 0.1 M citrate buffer at pH 6.0 for 20 minutes in a 95° C water bath. Slides were cooled, followed by sequential rinsing in PBS and 50mM Tris HCl, pH 7.6, 150 mM NaCl, Tween 20 (0.1%) (TBS-T). Endogenous peroxidase activity was quenched by incubation in TBS-T containing 3% hydrogen peroxide. Each incubation step was carried out at room temperature and was followed by three sequential washes (5 minutes each) in TBS-T. Sections were incubated in primary antibody, diluted 1:100 in TBS-T containing 1% ovalbumin and 1mg/ml sodium azide, followed by incubations with biotinylated secondary antibody for 15 minutes, peroxidase-labeled streptavidin for 15 minutes (LSAB-2 Agilent, K060911-8) and diaminobenzidine and hydrogen peroxide chromogen substrate (Dako Corp. Carpentaria CA, USA) for 10 minutes. The sections were lightly counterstained with Mayer’s hematoxylin solution and mounted with Clear mount (American Mastertech Scientific MMCO126). The antibody used was XPO1 (CRM1 H-300 sc-5595 Santa Cruz Biotechnology, Santa Cruz, CA, USA, dilution 1:100). The images were captured at 40× magnification with the Invitrogen EVOS FL Auto 2 Cell Imaging System.

### Immunofluorescence

10,000 cells/well were plated in an 8-well chamber slide (Nunc Lab-Tek 177445). The following day the cells were treated with selinexor or DMSO for 48 hours (UM-UC-3 and T24 were treated with 0.15 uM, J82 was treated with 0.25 uM, and TCCSUP was treated with 0.5 uM). Then the cells were gently washed twice with pre-warmed RPMI-1640 and were fixed with fresh 4% paraformaldehyde for 10 minutes at room temperature (RT). After fixation, the cells were washed with PBS + 0.3% Triton-X100 (PBS-Tx) for 10 minutes. Following the wash, the cells were incubated with OneBlock Western-CL Blocking Buffer (Genesee Scientific 20-313) for one hour at RT. The cells were then washed again with PBS-Tx for 5 minutes. Primary antibodies were diluted in OneBlock and the cells were incubated with the primary antibody overnight at 4° C in a humidified chamber (Cell Signaling Technology: RB 9309s, tubulin 3873s, tubulin 2125s; Novus Biologicals: p107 NBP2-33735; Santa Cruz Biotechnology: p130 sc-317, CRM1 sc-5595). Cells were rinsed twice with PBS and once with PBS-Tx for 5 minutes each. Secondary antibodies were diluted in OneBlock and cells were incubated with the secondary antibody for 1 hour at RT in the dark (ThermoFisher Scientific: Alexa Fluor 488 A11008, Alexa Fluor 647 A21235, Alexa Fluor 488 A11001, Alexa Fluor 647 A21244, Alexa Fluor 660 A21073). Following incubation, the cells were washed once with PBS-Tx and twice with PBS for 5 minutes each. Finally, the cells were mounted with ProLong Diamond Antifade Mountant with DAPI (Invitrogen P36971) and left to cure overnight at RT. The images were captured at 40x magnification with the Invitrogen EVOS FL Auto 2 Cell Imaging System and the images were quantified with Fiji [54].

### Caspase3/7 immunofluorescence assay

55,000 cells/well were plated in 6-well plates. The following day the cells were treated with either DMSO, 0.1 uM, or 1 uM selinexor for 72 hours. The drug containing media was removed and cells were treated with media (RPMI-1640 with 10% Fetal Bovine Serum and 1% Penicillin and Streptomycin) and CellEvent Caspase-3/7 Green (ThermoFisher Scientific R37111) (2 drops of CellEvent per mL of media) for 30 minutes and then imaged at 10× magnification with Invitrogen EVOS FL Auto 2 Cell Imaging System. The images were then quantified by counting the number of positive cells from 4 separate regions for each condition and cell line.

### Proliferation assays

For experiments assessing the growth of cells treated with various agents, cells were plated in a 24-well plate at 10,000 cells/well and the following day treated with drug or vehicle. Proliferation was assessed 72 hours later using Cell Counting Kit – 8 (CCK-8) (Dojindo CK04). All conditions were performed in triplicate and repeated at least twice. Data is displayed as the mean ± standard deviation.

For studies using siRB or non-targeting control and chemotherapeutic drugs, cells were plated 24 hours prior to transfection at 10,000–15,000 cells/well. 24 hours post transfection, cells were treated with drugs or vehicle. Proliferation was assessed 72 hours later using CCK-8. All conditions were performed in triplicate and repeated. Data is displayed as the mean ± standard deviation.

### Flow cytometry

T24 and UM-UC-3 cells were treated with selinexor or vehicle in triplicate. Following 72 hours of treatment, cells were harvested, ethanol fixed, and propidium iodide (PI) stained (Sigma-Aldrich 11348639001) for cell cycle analysis. All flow cytometry was carried out on a FACS Calibur flow cytometer (BD). Cell cycle data was analyzed using ModFit software. Data is displayed as the mean ± standard deviation.

### Colony formation assay

T24 and UM-UC-3 cells were plated in a 6-well plate at 1,500 cells/well. The following day, cells were treated with 0.1 uM selinexor or vehicle (DMSO) for 9 (J82), 10 (TCCSUP), 11 (UM-UC-3) or 12 (T24) days. -, -, - and -. Colonies were stained using crystal violet and photographed using an Alpha Innotech MultiImage II system. The analyses were performed in triplicate and repeated.

### Western blot

Cells were lysed in ice cold RIPA buffer supplemented with protease inhibitor cocktail (Sigma P 8340). Proteins were quantitated using a Bio-rad protein assay dye reagent concentrate (BIORAD 5000006) 10–50 ug of proteins were separated on 8,10 or 12 % acrylamide-bis SDS-PAGE and transferred to nitrocellulose membranes. Membranes were blocked in 5% milk in PBS-1% Tween 20 or with OneBlock (Genesee Scientific 20-313) and incubated with primary antibody overnight at 4° C. Membranes were washed with PBS-T 3 times and incubated with secondary antibody conjugated to HRP. Proteins were detected using ECL (Thermo Scientific 34096). The following antibodies were used: Cell Signaling Technology: PARP (9542s), RB (9309s), tubulin (2125s); Santa Cruz Biotechnology: p27 (sc-528), XPO1 (CRM1) (sc-5595), p130 (sc-317), cyclin A (sc-596), GAPDH (sc-32233), actin (sc-8432) and cdk2 (sc-163); Covance: nup62 (MMS-120P); Novus Biologicals: p107 (NBP2-33735); Bethyl: cyclin B1 (A305-000A). Cell fractionation of cellular lysates was performed with the NE-PER nuclear and cytoplasmic extraction reagents kit (Thermo Scientific # 78833) per manufacturer’s recommendation.

### Mouse studies

All experiments were conducted as approved by UC Davis IACUC Committee. 5-6-week old nu/nu athymic female mice were obtained from Jackson Laboratory. Suspensions of UM-UC-3 cells 10^6^ in 50% Matrigel solubilized basement membrane (Fisher #CB40234A) were subcutaneously injected to establish xenograft tumors. When palpable tumors were observed, mice were randomly assigned into the control or treatment group and gavaged with vehicle (0.6% Plasdone PVP-29/32 and 0.6% Poloxamer Pluronic F-68 in water) or selinexor (provided by Karyopharm) 15mg/kg three times per week for 24 days. The vehicle was prepared fresh weekly and the drug was prepared fresh for every use. Tumor volume of each tumor was measured 3 times per week length × width × depth. If tumors reach 2 cm in any direction or if they became ulcerated, the animal was euthanized. After euthanasia the tumors were removed and flash frozen. For western blot analysis, the tissue was lysed and homogenized in ice cold cell lysis buffer (50 mM Tris HCl, pH 7.4, 150 mM NaCl, and 1% NP-40, EGTA, EDTA, a cocktail of protease inhibitors (Thermo Scientific 78410), and phosphatase inhibitors: 20 mM β-glycerol phosphate, 1 mM Na-orthovanadate, and 10 mM NaF).

### Statistics

A two tailed, unpaired equal variance student’s *T*-test was used to assess differences between samples with equal variance and two-tailed unpaired unequal variance was used for samples with unequal variance. A *p* < 0.05 was accepted as significant. The combination index (CI) was calculated using the Bliss Independence model equation: CI = effect of drug A + effect of drug B – (effect of drug A X effect drug B/effect of combined AB. CI = 1 indicates an additive interaction, CI ˂ 1 indicates synergy and a CI ˃ 1 indicates antagonism. GraphPad Prism 6 was used to conduct Kaplan–Meier analysis.

## SUPPLEMENTARY MATERIALS FIGURES



## Abbreviations

XPO1: Exportin-1/chromosome region maintenance 1
RB: retinoblastoma
CDK: cyclin dependent kinase
NMIBC: non-muscle invasive bladder cancer
MIBC: muscle invasive bladder cancer
GC: gemcitabine and cisplatin
MVAC: methotrexate, vinblastine, doxorubicin, and cisplatin
SINE: selective inhibitor of nuclear export
IF: immunofluorescence
DMSO: dimethyl sulfoxide
PARP: Poly (ADP-ribose) polymerase
Wt: wildtype
PDX: patient derived xenograft
