# Supplementary material for: XPO1 inhibition by selinexor induces potent cytotoxicity against high grade bladder malignancies
Source: Oncotarget. 2018 Oct 2;9(77):34567–81. doi: 10.18632/oncotarget.26179 (PMC6195388; doi:10.18632/oncotarget.26179)
Supplement: Supplementary file 1 [file oncotarget-09-34567-s001.pdf]

XPO1 inhibition by selinexor induces potent cytotoxicity against high grade bladder malignancies

SUPPLEMENTARY MATERIALS

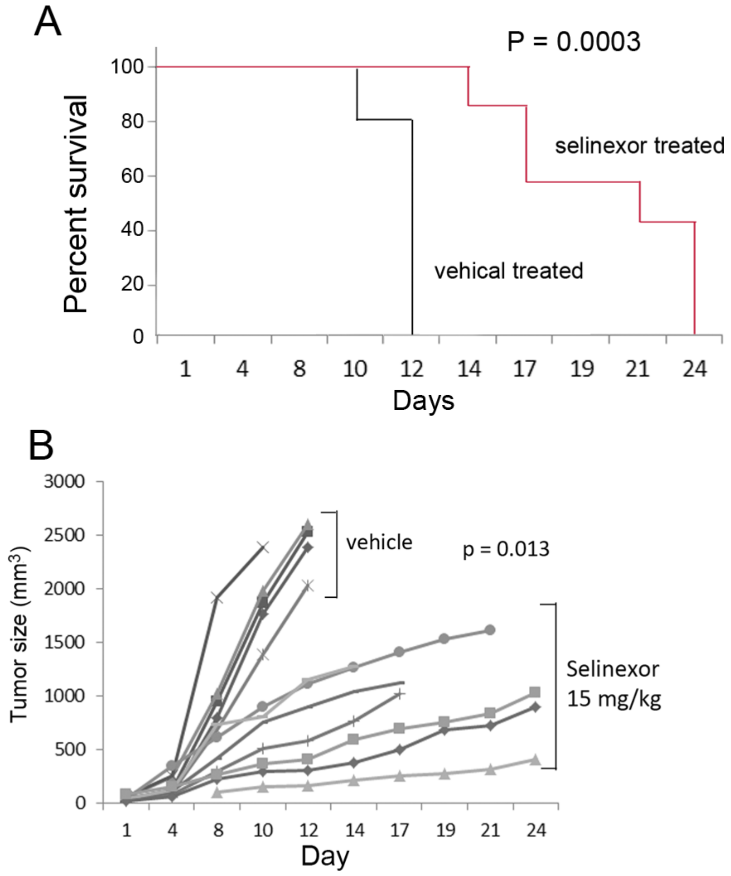

Supplementary Figure 1: (**A**) Kaplan–Meier survival analysis. (**B**) Growth curves of individual tumors.

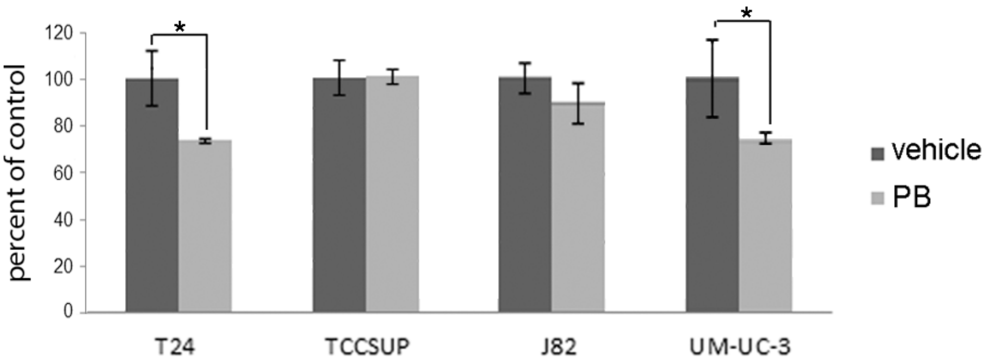

Supplementary Figure 2: CDK4/6 inhibitor Palbociclib reduces viability of T24 and UM-UC-3 cells. Cells were treated with 0.5 µm PB or vehicle control for 3 d.
